# Supplementary material for: A Common Allele in FGF21 Associated with Sugar Intake Is Associated with Body Shape, Lower Total Body-Fat Percentage, and Higher Blood Pressure
Source: Cell Rep. 2018 Apr 10;23(2):327–36. doi: 10.1016/j.celrep.2018.03.070 (PMC5912948; doi:10.1016/j.celrep.2018.03.070)
Supplement: Document S1. Tables S1–S6 [file mmc1.pdf]

Cell Reports, Volume *23*

Supplemental Information

A Common Allele in FGF21 Associated with Sugar Intake Is Associated with Body Shape, Lower Total Body-Fat Percentage, and Higher Blood Pressure

Timothy M. Frayling, Robin N. Beaumont, Samuel E. Jones, Hanieh Yaghootkar, Marcus A. Tuke, Katherine S. Ruth, Francesco Casanova, Ben West, Jonathan Locke, Seth Sharp, Yingjie Ji, William Thompson, Jamie Harrison, Amy S. Etheridge, Paul J. Gallins, Dereje Jima, Fred Wright, Yihui Zhou, Federico Innocenti, Cecilia M. Lindgren, Niels Grarup, Anna Murray, Rachel M. Freathy, Michael N. Weedon, Jessica Tyrrell, and Andrew R. Wood

SUPPLEMENTARY INFORMATION

Supplementary table 1. Details of UK Biobank participants taking Food frequency questionnaire, related to Table 1.

Demographic	Completed FFQ	Not completed FFQ	P		Demographic	Completed FFQ more than once	Completed FFQ once only	P
N	188,060	120,185			N	117,624	70,436	
Age at recruitment (SD)	56.8 (7.8)	56.3 (8.1)	$<1x10^{-15}$		Age at recruitment (SD)	56.8 (7.8)	56.8 (8.0)	0.58
Male, N(%)	84,976 (45.2)	58,128 (48.4)	$<1x10^{-15}$		Male, N(%)	52,298 (44.5)	32,678 (46.4)	$<1x10^{-15}$
Mean BMI (SD)	26.9 (4.6)	27.6 (4.7)	$<1x10^{-15}$		Mean BMI (SD)	26.7 (4.6)	27.2 (4.7)	$<1x10^{-15}$
Mean body fat percentage (SD)	30.8 (8.4)	31.3 (8.4)	$<1x10^{-15}$		Mean body fat percentage (SD)	30.6 (8.4)	31.1 (8.4)	$<1x10^{-15}$
Mean systolic blood pressure (SD)	142 (23)	143 (24)	$<1x10^{-15}$		Mean systolic blood pressure (SD)	142 (23)	143 (24)	$2x10^{-8}$
Mean waist circumference (SD)	89.0 (13.2)	90.9 (13.4)	$<1x10^{-15}$		Mean waist circumference (SD)	88.6 (13.3)	89.9 (13.4)	$<1x10^{-15}$
Mean hip circumference (SD)	103 (9)	104 (9)	$<1x10^{-15}$		Mean hip circumference (SD)	103 (9)	103 (9)	$<1x10^{-15}$
Mean WHR (SD)	0.86 (0.09)	0.87 (0.09)	$<1x10^{-15}$		Mean WHR (SD)	0.86 (0.09)	0.87 (0.09)	$<1x10^{-15}$
Mean ACR (SD)	1.62 (2.2)	1.57 (2.4)	0.001		Mean ACR (SD)	1.63 (2.21)	1.61 (2.35)	0.56
T2D, N (%)	5,045 (2.7)	3,590 (3.0)	$2x10^{-12}$		T2D, N (%)	2,978 (2.5)	2,067 (2.9)	$3x10^{-6}$
CAD, N(%)	12,262 (6.5)	9,452 (7.9)	$<1x10^{-15}$		CAD, N(%)	7,066 (6.0)	5,196 (7.4)	$<1x10^{-15}$

Supplementary table 2. Details of the association between rs838133 and anthropometric traits in men and women separately, related to Table 2.

Anthropometric trait	Sex	BETA	SE	P
Body fat percentage	men	-0.00750807	0.003019	0.012
Body fat percentage	women	-0.00950441	0.002766	0.0012
BMI	men	-0.00443286	0.003055	0.11
BMI	women	-0.00582809	0.0028	0.056
Hip circumference	men	-0.0134019	0.003065	8.9E-06
Hip circumference	women	-0.0135767	0.002797	2.1E-06
Waist circumference	men	-0.00466646	0.003066	0.082
Waist circumference	women	-0.00519281	0.002795	0.063
WHRadjBMI	men	0.0114921	0.003166	0.00022
WHRadjBMI	women	0.00632038	0.002825	0.048
WHR	men	0.00575562	0.003136	0.061
WHR	women	0.00991502	0.002817	0.0004
Height	men	-0.0105833	0.002691	0.00053
Height	women	-0.0125656	0.002426	5.4E-08

Supplementary table 3. Associations between the minor A allele at rs838133 and anthropometric and metabolic traits in a subset of individuals with food frequency questionnaire data available, before and after adjusting for percentage macronutrient intake, related to Table 2.

Adjusting for percentage carbs, fat and protein					Unadjusted in same number		
**Trait**	**N**	**Beta SD or OR**	**SE or 95%CI**	**P**	**Beta SD or OR**	**SE or 95%CI**	**P**
Body fat %	148609	-0.0049	0.0038	0.19	-0.0088	0.0038	0.021
BMI	150514	-0.0042721	0.0037	0.25	-0.0084	0.0037	0.025
Hip circumference	150792	-0.0093	0.0037	0.012	-0.0129	0.0037	$5x10^{-4}$
Waist circumference	150804	-0.0024	0.0037	0.51	-0.0059	0.0037	0.12
WHRadjBMI	150483	0.0086	0.0037	0.021	0.0089	0.0037	0.017
WHR	150778	0.0053	0.0037	0.16	0.0035	0.0038	0.35
Height	150761	-0.0082	0.0037	0.029	-0.0077	0.0037	0.04
ACR	146872	0.0074	0.0038	0.049	0.0083	0.0038	0.027
BP meds**	27453/122939	1.02	1.00-1.04	0.039	1.02	1.00-1.04	0.07
CAD**	9871/104533	1	0.97-1.03	0.92	1	0.97-1.03	0.88
DBP	150477	0.0113	0.0037	0.002	0.0108	0.0037	0.004
SBP	150690	0.0126	0.0037	0.0007	0.0125	0.0037	0.0007
Hypertension**	75611/74683	1.03	1.01-1.04	0.0007	1.03	1.01-1.04	0.0009
T2D**	4079/144599	0.96	0.92-1.01	0.11	0.95	0.91-1.00	0.036

Supplementary Table 4. Association between rs439523, in LD with rs838133 ($r^2 = 0.62$), and *FGF21* expression in human liver. Within three liver eQTL data sets, linear regression was used to model *FGF21* expression levels with adjustment for relevant covariates. Results from the three liver datasets were combined by meta-analysis. *FGF21* expression level was determined using microarray and only included patients of European ancestry. The data was coded such that a negative beta means that as the number of minor alleles increases there is a decrease in *FGF21* expression, related to experimental procedures.

				rs439523		
**Dataset**	**n**	**Expression**	**Genotyping**	**P-value**	**Beta**	**PMID**
Dataset 1	164	Agilent-014850 Whole Human Genome 4x44K gene expression (NCBI GEO accession: GSE25935)	Illumina Human610-Quad v1.0 BeadChip (NCBI GEO accession: GSE26105)	0.0811	-0.0141	21637794
Dataset 2	286	Agilent Technologies (NCBI GEO accession: GSE9588)	Affymetrix GeneChip Human Mapping 500k genotyping microarray	0.4700	-0.0055	18462017
Dataset 3	581	Agilent Technologies (NCBI GEO accession: GSE9588)	HumanHap 650Y	0.9643	-0.0002	21602305
Meta	1031			0.3076	$t_{meta} = -1.0202$	

Supplementary Table 5. "Phenome wide association study" in 451,099 individuals. Associations between the rs838133 variant and 82 traits in UK Biobank. Emboldened rows highlight associations reaching false discovery rates of 1% or less (p<0.012), related to experimental procedures.

Trait	BETA	SE	P
**Systolic blood pressure**	**0.0120336**	**0.00184876**	**2E-10**
**Hip circumference**	**-0.0121696**	**0.00203943**	**8E-10**
**Height**	**-0.0103052**	**0.00171557**	**2.4E-09**
**Albumin creatine ratio, continous**	**0.0121483**	**0.00208866**	**6.2E-09**
**Hypertension**	**0.00506564**	**0.000988123**	**0.00000066**
**Fat free mass in the arm (bioimpedence)**	**-0.00564884**	**0.00124359**	**0.0000031**
**Diastolic blood pressure**	**0.00920308**	**0.001977**	**0.0000038**
**Body fat mass (bioimpedence)**	**-0.00907592**	**0.00197836**	**0.0000039**
**Waist hip ratio adjusted for BMI**	**0.0100678**	**0.0022142**	**0.000004**
**Blood pressure medication**	**0.00342205**	**0.00083121**	**0.000039**
**Body fat mass % (bioimpedence)**	**-0.00602927**	**0.0015396**	**0.00013**
**Body fat free mass % (bioimpedence)**	**-0.00428308**	**0.00122452**	**0.00024**
**Chronotype (morning or evening person)**	**-0.00987515**	**0.00278196**	**0.00058**
**IPAQ activity**	**-0.00728667**	**0.0022639**	**0.0013**
**Waist hip ratio**	**0.00676474**	**0.00220517**	**0.0022**
**Father's age at death**	**-0.00750113**	**0.00252796**	**0.003**
**Birth weight**	**-0.00855478**	**0.00283002**	**0.0032**
**Lost weight in the last year**	**-0.00312441**	**0.00105482**	**0.0032**
**Limb fat mass (bioimpedence)**	**-0.00379891**	**0.00134292**	**0.0061**
**Psoriasis**	**0.000840724**	**0.000334925**	**0.0091**
**Osteoporosis**	**0.000717142**	**0.000280175**	**0.0095**
**Age first child born**	**0.00802482**	**0.00312645**	**0.0096**
Albumin creatinine ratio using cut off of <=3.5	0.00150966	0.000608407	0.014
diverticular disease ICD10	0.00121587	0.000534468	0.02
Waist circumference	-0.00391468	0.00180551	0.022
Number of children fathered	-0.00702426	0.00317092	0.027
Length of menstrual cycle	0.0167682	0.00771689	0.039
Age at menarche	-0.00906782	0.00447201	0.042
Coronary artery disease	0.00145283	0.000728024	0.043
undersleeper	0.00201585	0.000991189	0.046
Ectopic pregnancy	-0.000234989	0.000119476	0.049
Major Depresssion	-0.000304361	0.000164135	0.064
Body mass index	-0.00349568	0.00213527	0.1
Ovarian cysts	0.000603848	0.000367178	0.1
Fibroids	0.000736002	0.000497518	0.14
Arterial Stiffness	-0.00526339	0.00363522	0.16
Number of births	-0.00413257	0.00287968	0.16
Major depressive disorder	0.000998134	0.00073579	0.17
Low grip strength in >=60 year olds	0.00143402	0.00113557	0.2
Number of pregnancies - women	-0.00341784	0.00294489	0.25

Trait	BETA	SE	P
Napping	0.00142163	0.00125651	0.25
Infertile	0.00015435	0.000143199	0.28
Mother's age at death	-0.00300551	0.00279656	0.28
Migraine	-0.000394451	0.000378319	0.29
Still birth	0.000483813	0.000455413	0.29
First child preterm?	0.000711064	0.000695798	0.31
preterm birth	-0.000597964	0.000604261	0.32
First child's birthweight	-0.00302311	0.00331566	0.34
Myocardial infarction	0.000288971	0.000325801	0.38
Major depressive disorder - recurrent	0.000572699	0.000674668	0.4
Reproductive life span	-0.0029088	0.00427444	0.45
Hours slept (excluding <3, >14)	-0.00168035	0.00233723	0.48
Major depressive disorder - single episode	0.000278265	0.000403138	0.49
Polycystic ovarian syndrome	-9.72881E-05	0.000139946	0.49
Fried index: 0-2 (not frail) 3-5 (frail)	-0.000420167	0.000659628	0.52
Spontaneous miscarriage	0.000751262	0.00120625	0.53
Gastro-oesophageal reflux syndrome	-0.00046589	0.000763007	0.55
Gestational diabetes	-0.000140713	0.000237492	0.55
Oversleeper	0.000389081	0.000751036	0.59
Have at least one of gord, h_hernia, Barrett's c	-0.000447127	0.000864513	0.6
Osteoarthritis	-0.000301594	0.000752518	0.71
Breast Cancer	0.000305281	0.000659173	0.71
Ovarian cancer	7.19151E-05	0.000206199	0.73
Menopause before age 45, age >50 as controls	-0.000211173	0.00133303	0.75
endometriosis	0.000120854	0.000365912	0.75
number of pregnancies lost	-0.000926449	0.00297796	0.76
Cancer registry yes no	0.000262795	0.000727278	0.77
Insomnia (3 categories)	0.000421846	0.00153804	0.78
Age at menopause < 40 years	-0.000089953	0.000338761	0.79
sedentary time	-0.000583996	0.00209313	0.79
Type 2 diabetes strict defintion	9.62441E-05	0.000385218	0.79
Snorer	-0.000241752	0.00104514	0.8
number of still births	-0.000587357	0.00297666	0.84
Gain weight in the last year	-0.000237803	0.00110528	0.85
Premature ovarian insufficiency (Cases are nat	4.00769E-06	0.00168513	0.85
number of miscarriages	0.000505664	0.00297698	0.87
Uterine cancer	3.04602E-05	0.000241324	0.9
Natural age at menopause	0.000414949	0.00432607	0.95
Bone mineral density	-0.000632625	0.00190941	0.96
preeclampsia	-1.04041E-05	0.000275402	0.97
Menorrhagia	2.36807E-06	0.000229386	0.99

Supplementary table 6. Validation of associations of physical activity and chronotype in up to 96,034 individuals with objective accelerometer based measures, related to experimental procedures.

Accelerometer derived activity trait	BETA	SE	P
Proportion of activity over 100mg (moderate to vigorous)	-0.00769687	0.00468483	0.098
Proportion of activity over 40mg (non sedentary)	-0.0101416	0.00468731	0.029
Proportion of activity under 40mg (non sleeping sedentary)	0.0104298	0.00468728	0.025
Midpoint sleep*	0.0114798	0.00494578	0.023

*based on timing of average of least active 5 hours during 24 hour daily cycle.
