# Supplementary material for: A Common Allele in FGF21 Associated with Sugar Intake Is Associated with Body Shape, Lower Total Body-Fat Percentage, and Higher Blood Pressure
Source: Cell Rep. 2018 Apr 10;23(2):327–36. doi: 10.1016/j.celrep.2018.03.070 (PMC5912948; doi:10.1016/j.celrep.2018.03.070)
Supplement: Document S2. Article plus Supplemental Information [file mmc2.pdf]

Cell Reports

A Common Allele in FGF21 Associated with Sugar Intake Is Associated with Body Shape, Lower Total Body-Fat Percentage, and Higher Blood Pressure

Graphical Abstract

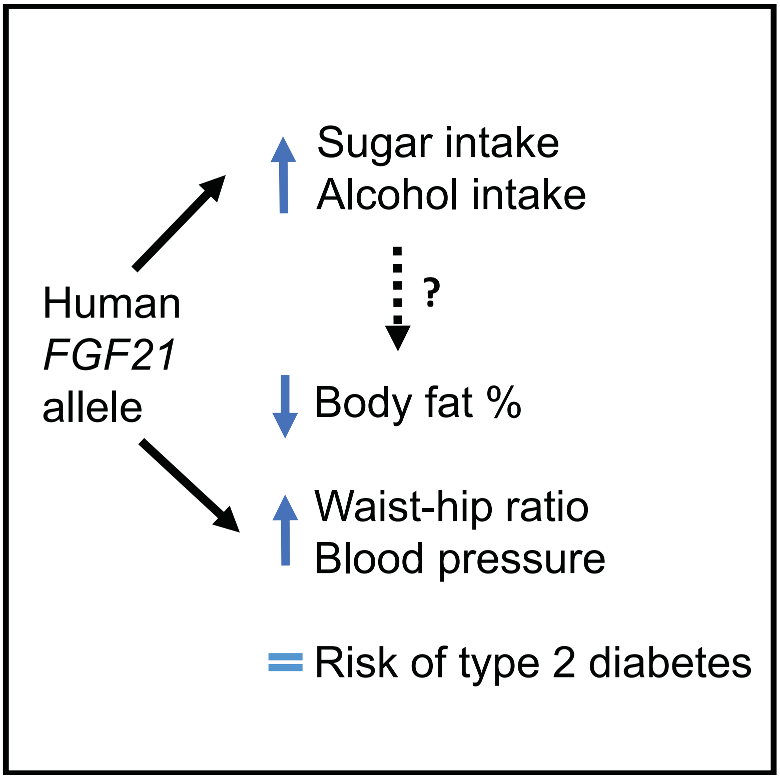

Authors

Timothy M. Frayling, Robin N. Beaumont, Samuel E. Jones, ..., Michael N. Weedon, Jessica Tyrrell, Andrew R. Wood

Correspondence

t.m.frayling@exeter.ac.uk

In Brief

Drugs targeting the hormone FGF21 may have beneficial health effects. Variations in human DNA in the FGF21 gene provide an indication of what those effects may be. Here, we show that variation in the FGF21 gene is associated with higher blood pressure and altered body shape, despite lower total body-fat percentage.

Highlights

- A human allele in *FGF21* is associated with higher alcohol as well as sugar intake
- Phenotypes associated with this allele likely mimic those of lower FGF21 function
- The human allele in *FGF21* is associated with lower total body-fat percentage
- The human allele in *FGF21* is associated with higher blood pressure and waist-hip ratio

Frayling et al., 2018, Cell Reports *23*, 327–336
April 10, 2018 © 2018 The Authors.
https://doi.org/10.1016/j.celrep.2018.03.070

CellPress

Cell Reports
Report

OPEN ACCESS
CellPress

A Common Allele in FGF21 Associated with Sugar Intake Is Associated with Body Shape, Lower Total Body-Fat Percentage, and Higher Blood Pressure

Timothy M. Frayling,[1,10,*] Robin N. Beaumont,[1] Samuel E. Jones,[1] Hanieh Yaghootkar,[1] Marcus A. Tuke,[1] Katherine S. Ruth,[1] Francesco Casanova,[1,2] Ben West,[1] Jonathan Locke,[1] Seth Sharp,[1] Yingjie Ji,[1] William Thompson,[1] Jamie Harrison,[1] Amy S. Etheridge,[3] Paul J. Gallins,[4] Dereje Jima,[4] Fred Wright,[4] Yihui Zhou,[4] Federico Innocenti,[3] Cecilia M. Lindgren,[5,6,7] Niels Grarup,[8] Anna Murray,[1] Rachel M. Freathy,[1] Michael N. Weedon,[1] Jessica Tyrrell,[1,9] and Andrew R. Wood[1]

[1]Genetics of Complex Traits, University of Exeter Medical School, RILD Level 3, Royal Devon & Exeter Hospital, Barrack Road, Exeter EX2 5DW, UK
[2]Diabetes and Vascular Research Centre, University of Exeter Medical School, Royal Devon & Exeter Hospital, Barrack Road, Exeter EX2 5DW, UK
[3]The University of North Carolina, Eshelman School of Pharmacy and Center for Pharmacogenomics and Individualized Therapy, Chapel Hill, NC, USA
[4]Bioinformatics Research Center, North Carolina State University, Raleigh, NC, USA
[5]Big Data Institute at the Li Ka Shing Centre for Health Information and Discovery, University of Oxford, Oxford, UK
[6]Wellcome Trust Centre for Human Genetics, University of Oxford, Oxford, UK
[7]Program in Medical and Population Genetics, Broad Institute, Cambridge, MA, USA
[8]Novo Nordisk Foundation Center for Basic Metabolic Research, Faculty of Health and Medical Sciences, University of Copenhagen, Copenhagen, Denmark
[9]European Centre for Environment and Human Health, University of Exeter Medical School, The Knowledge Spa, Royal Cornwall Hospital, Truro TR1 3HD, UK
[10]Lead Contact
*Correspondence: t.m.frayling@exeter.ac.uk
https://doi.org/10.1016/j.celrep.2018.03.070

SUMMARY

Fibroblast growth factor 21 (FGF21) is a hormone that has insulin-sensitizing properties. Some trials of FGF21 analogs show weight loss and lipid-lowering effects. Recent studies have shown that a common allele in the *FGF21* gene alters the balance of macronutrients consumed, but there was little evidence of an effect on metabolic traits. We studied a common *FGF21* allele (A:rs838133) in 451,099 people from the UK Biobank study, aiming to use the human allele to inform potential adverse and beneficial effects of targeting FGF21. We replicated the association between the A allele and higher percentage carbohydrate intake. We then showed that this allele is more strongly associated with higher blood pressure and waist-hip ratio, despite an association with lower total body-fat percentage, than it is with BMI or type 2 diabetes. These human phenotypes of variation in the *FGF21* gene will inform research into FGF21's mechanisms and therapeutic potential.

INTRODUCTION

FGF21 is a hormone secreted primarily by the liver whose multiple functions include signaling to the paraventricular nucleus of the hypothalamus to suppress sugar and alcohol intake (von Holstein-Rathlou et al., 2016; Talukdar et al., 2016a), stimulating glucose uptake by adipocytes (Kharitonenkov et al., 2005) and acting as an insulin sensitizer (Berglund et al., 2009; Bondurant et al., 2017). These features and several other lines of evidence have prompted the development of FGF21-based therapies as potential treatments for obesity and type 2 diabetes, with consistent effects on triglyceride lowering, some effects on weight loss, but little effect on glucose tolerance (Kharitonenkov and DiMarchi, 2017; Reitman, 2013). An early trial showed lipid-lowering effects in people with type 2 diabetes and obesity, but there was only suggestive evidence for effects on weight and glucose tolerance (Gaich et al., 2013). A recent study suggested that FGF21 analogs may alter blood pressure in humans (Kim et al., 2017), although changes in blood pressure were not observed in a previous trial (Talukdar et al., 2016b). Pre-clinical evidence of FGF21's potential role in metabolism includes resistance to diet induced obesity in mice overexpressing FGF21 (Kharitonenkov et al., 2005) and improved glucose tolerance in obese mice through administration of recombinant FGF21 (Kharitonenkov et al., 2005). Subsequent studies have confirmed these findings in mice (Coskun et al., 2008; Bondurant et al., 2017) and shown similar effects in non-human primates, including improvement of glucose tolerance and slight weight loss in diabetic rhesus monkeys (Kharitonenkov et al., 2007), but other studies are less conclusive (Kharitonenkov and DiMarchi, 2017).

This is an open access article under the CC BY license (http://creativecommons.org/licenses/by/4.0/).

Recent studies have shown that FGF21 affects the balance of macronutrients consumed. Studies in mice and non-human primates show that genetically and pharmacologically raising FGF21 levels suppresses sugar and alcohol intake (Talukdar et al., 2016a; von Holstein-Rathlou et al., 2016). Three human genetic studies have shown that a common allele at rs838133 (A/G, minor allele frequency = 44.7%), which results in a synonymous change to the first exon of *FGF21*, is associated with higher carbohydrate and lower protein and fat intake, with no effect on total calorie intake (Chu et al., 2013; Soberg et al., 2017; Tanaka et al., 2013). Soberg et al. (2017) showed that the carbohydrate preference was specific to sugary products and may also increase alcohol intake. These findings are consistent with data from animal studies showing that FGF21 signals to reward centers in the brain (Talukdar et al., 2016a; von Holstein-Rathlou et al., 2016). The human genetic studies found no detectable effect on the risk for type 2 diabetes and only nominal evidence for an effect on BMI (Soberg et al., 2017).

Human genetic germline variation provides a potentially very informative way of assessing the likely efficacy and adverse effects of therapies. Importantly, the effect size of a genetic association with a given trait is unrelated to the likelihood that the relevant gene product might be an adequate drug target. A statistically robust association suggests that pharmacological manipulation of the target gene to stronger gain or loss of function will alter the trait to a larger extent than that conferred by the naturally occurring allele. Examples include alleles in the *PCSK9*, *NPC1L1*, and *HMGCR* genes, all associated with lower low-density lipoprotein (LDL) cholesterol and lower risk for atherosclerotic heart disease. These alleles are also associated with very subtly higher risk for type 2 diabetes, with odds ratios of less than 1.06 (Ference et al., 2016; Lotta et al., 2016; Schmidt et al., 2017; Swerdlow et al., 2015) but add genetic evidence to that from trials showing that such LDL lowering increases the risk for type 2 diabetes (Frayling, 2015; Swerdlow et al., 2015).

Here we aimed to extend the characterization of the phenotypes associated with the variant in *FGF21* using 451,000 individuals from the UK Biobank. We reasoned that genotype-phenotype associations of *FGF21* would generate hypotheses for developers of FGF21-based therapies about their potential beneficial and adverse effects. The use of human genetic information is increasingly viewed as an important step to inform drug development (Hurle et al., 2016). Human genotype-phenotype associations will also inform experimental studies of FGF21 function. We first replicated the association between the minor allele at rs838133 and higher carbohydrate and lower protein and fat intake. We then provide conclusive evidence that the same allele increases alcohol intake, consistent with findings from animal studies and meaning the allele's effects on human nutrient intake mimic perfectly those seen in FGF21 model organisms. We identified several associations with metabolic and anthropometric traits (all with statistical confidence $< 6 \times 10^{-5}$), most likely not detected before because of limited power: the *FGF21* rs838133 A allele is associated with stronger effects on higher waist-hip ratio and higher blood pressure, despite an association with lower total body-fat percentage, than its effects on BMI, and has no detectable effect on type 2 diabetes.

RESULTS

The Minor Allele at FGF21 rs838133 Is Associated with Higher Sugar and Alcohol Intake and Lower Protein and Fat Intake

We first investigated the previously described associations between the *FGF21* rs838133 variant and macronutrient intake, coffee and alcohol intake, and smoking. We used data including that derived from a food frequency questionnaire (FFQ) completed by up to 176,994 UK Biobank participants from among 451,099 we defined as of European ancestry. We used a p value of 0.0005 as an equivalent of p = 0.05 given the 100 tests performed. In Table 1 we show how each copy of the minor A allele was associated with higher self-reported estimates of carbohydrate and alcohol intake and lower fat and lower protein intake. All these associations reached

Table 1. Associations between the Minor A Allele at rs838133 and Self-Reported Diet and Smoking Measures in the UK Biobank

Quantitative Diet Outcome	n	Beta Raw (units)	Beta (SD)	SE	p
Alcohol units	341,878	0.015 (units)	0.0147	0.0022	4×10^{-11}[a]
Total energy	176,994	−9.623 (KJ)	−0.0036	0.0033	0.28
% protein	176,989	−0.108 (%)	−0.0291	0.0034	3×10^{-17}[a]
% carbs	176,989	0.206 (%)	0.0244	0.0035	2×10^{-12}[a]
% fat	176,989	−0.196 (%)	−0.0281	0.0347	4×10^{-16}[a]
Fizzy drink consumption	176,994	−0.001	−0.0028	0.0035	0.41
Cups of coffee per day[b]	299,908	−0.014	−0.0076	0.0027	0.005
Binary Outcomes	Number Yes (No)		OR	95% CI	
Current smoker (yes or no)	35,946 (204,252)	NA	0.997	(0.980–1.013)	0.68
Ever smoker (yes or no)	170,388 (204,252)	NA	0.991	(0.981–1.000)	0.06
Coffee (yes or no)	299,908 (79,235)	NA	0.987	(0.976–0.998)	0.025

Macronutrient intake and fizzy drink intake data are based on FFQs completed by up to 176,994 individuals between one and five times. Fizzy drink intake includes calorie-free drinks. Other data are based on questionnaires completed at baseline collection. Effect sizes are SD per A allele. "Beta raw" refers to effect size based on untransformed variable. CI, confidence interval; NA, not applicable; OR, odds ratio.[a]Associations reaching p < 0.0005, a correction for the approximately 100 tests performed.
[b]Within coffee drinkers (>10 cups per day collapsed into one group).

genome-wide levels of statistical confidence. These effects imply very strongly that the rs838133 A allele (population frequency = 45%) results in lower *FGF21* function because genetic and pharmacological lowering of FGF21 in animal models, including non-human primates, has exactly the same effect on carbohydrate and alcohol preferences (Talukdar et al., 2016a; von Holstein-Rathlou et al., 2016). We present all results aligned to this putative lower function allele, but the opposite directions can be interpreted as the putative effects of higher FGF21 function. The largest effect on macronutrient intake was with carbohydrate intake, where each A allele raised percentage intake by 0.21%. There was no detectable effect on total energy intake. Any effects on coffee consumption and smoking were minimal in comparison. In Table S1 we show the summary characteristics of people in the UK Biobank, including those completing the FFQ and those not completing it.

The Minor Allele at FGF21 rs838133 Is Associated with Lower Levels of Body Fat as a Percentage and Paradoxically Higher Waist-Hip Ratio

Previous studies reported only nominal evidence for associations between rs838133 and anthropometric measures. Using data from 451,099 UK Biobank participants, we showed that the minor rs838133 allele was associated with lower total body-fat percentage (−0.051% per allele, $p = 5 \times 10^{-5}$), equivalent to 20 g in a 100 kg person with 40% fat. Lower levels of body fat are usually associated with a lower waist-hip ratio (WHR), but paradoxically, the *FGF21* allele was associated with higher WHR before and after adjusting for BMI ($p = 2 \times 10^{-7}$ after adjusting for BMI). The strongest association, reaching genome-wide levels of statistical confidence, was with lower hip circumference ($p = 7 \times 10^{-13}$), for which each allele was associated with an approximately 1.0 mm difference. There was only nominal evidence of an effect on BMI. The minor A allele was also associated with shorter stature (also by ∼1 mm per allele), but this effect on reduced growth did not account for the smaller hip circumference (Table 2). Each of these associations with anthropometric traits was consistent with previously published GWAS data from the GIANT consortium (Table 2). Unlike many other variants altering WHR, the effects were very similar in men and women (Table S2).

The Minor Allele at FGF21 rs838133 Is Associated with Higher Blood Pressure and Altered Lipid and Liver Enzyme Levels, but Not Type 2 Diabetes or Heart Disease

The A allele at *FGF21,* associated with lower total body-fat percentage and higher WHR was also associated with higher blood pressure, hypertension, and blood pressure medication use. The effect sizes were not reduced after correcting for self-reported alcohol intake, smoking, and salt intake (Table 2). There was no association with coronary artery disease or type 2 diabetes, and the confidence intervals for any effect of the variant on type 2 diabetes ranged from a 0.99–1.03 odds ratio. We also noted an association with albumin/creatine ratio (ACR) at genome-wide levels of statistical confidence (Table 2). The largest effect was with systolic blood pressure, for which each A allele raised systolic blood pressure by 0.29 mm Hg; this association reached genome-wide levels of statistical confidence. Where data were available from existing GWAS (for blood pressure, coronary artery disease, and type 2 diabetes), a meta-analysis of existing associations and those in UK Biobank strengthened the statistical confidence of the findings (Table 2).

Finally, we examined the association between the *FGF21* rs838133 allele and relevant glycemic and liver and lipid markers that were not available in the UK Biobank but were available in published genome-wide association study (GWAS) data (Table 3). The allele associated with higher sugar intake and lower body-fat percentage was also associated with higher LDL cholesterol and gamma-glutamyl transpeptidase (GGT) levels and lower alkaline phosphatase (ALP) levels in existing GWAS data (Chambers et al., 2011; Willer et al., 2013). There was a nominal association with triglycerides. The directions of the associations with GGT and ALP were consistent with an effect of higher alcohol intake and lower protein and fat intake, respectively, although details of alcohol and macronutrient intake are not available in these studies to confirm this as the cause of the liver function test associations.

Adjusting the FGF21 Anthropometric and Metabolic Associations for Macronutrient Intake

We next reasoned that the associations with anthropometric and metabolic measures could be secondary to the *FGF21* minor allele's effect on altered macronutrient intake. To test this possibility, we performed additional analyses adjusting for the measures of percentage carbohydrate, protein, and fat intake derived from self-report FFQs available in a subset of individuals. These analyses showed two things. First, there was no evidence that the *FGF21* allele's effect on macronutrient intake leads to the effects on body shape and blood pressure. The associations with WHR adjusted for BMI, diastolic and systolic blood pressure, and hypertension (and hypertension medication) were very similar in both adjusted and unadjusted analyses. Second, there was a trend toward the *FGF21* allele's effect on macronutrient intake contributing to the differences in overall BMI and body-fat percentage. Although the differences between adjusted and unadjusted analyses did not reach $p < 0.05$, the adjusted effect sizes were approximately three-quarters of the unadjusted effect sizes (Table S3).

rs838133 Association with Liver FGF21 Expression

To provide additional insight into the *FGF21* rs838133 associations, we next tested its association with liver gene expression in a meta-analysis using 1,031 individuals from three hepatic expression quantitative trait locus (eQTL) studies. Because rs838133 was not included on the genotyping platforms for these studies and was in a region with low imputation quality, rs439523 ($r^2 = 0.62$ in genetic Europeans) was used as a proxy. There was no evidence of association with *FGF21* expression (Table S4).

Phenome-wide Association Study Shows Suggestive Associations of FGF21 Allele with Circadian Rhythm and Physical Activity

FGF21 has multiple metabolic effects, possibly all linked to a role as a "global starvation signal" (Bookout et al., 2013), and studies

Table 2. Associations between the Minor Allele at rs838133 and Anthropometric and Metabolic Traits in UK Biobank and Published GWAS Data Where Available

	UK Biobank					Published GWAS				Meta-analysis			
Anthropometric trait	n	Beta Raw	Beta SD or OR	SE or 95% CI	p	n	Beta SD or OR	SE or 95% CI	p	Beta SD	SE or 95% CI	p	GWAS Reference
Body-fat %	443,000	−0.051	−0.0060	0.0015	0.00013	69,791	−0.0045	0.0063	0.48	−0.0059	0.0015	0.00005[a]	Lu et al. (2016)
BMI	449,325	−0.017	−0.0035	0.0021	0.1	223,372	−0.0096	0.0042	0.022	−0.0047	0.0019	0.012	Locke et al. (2015)
Hip circ.	450,276	−0.11	−0.0122	0.0020	8×10^{-10}	134,725	−0.0210	0.0051	0.000049	−0.0134	0.0019	7×10^{-13}[a]	Shungin et al. (2015)
Hip circ.[b]	450,276	−0.09	−0.0110	0.0023	0.0000019	NA	NA	NA	NA	NA	NA	NA	NA
Waist circ.	450,323	−0.05	−0.0039	0.0018	0.022	143,054	−0.0120	0.0048	0.01	−0.0049	0.0017	0.004	Shungin et al. (2015)
WHR adj. for BMI	449,216	NA	0.0101	0.0022	0.000004	129,682	0.0120	0.0048	0.014	0.0104	0.002	2×10^{-7}[a]	Shungin et al. (2015)
WHR	449,216	NA	0.0068	0.0022	0.0022	133,877	0.0060	0.0048	0.21	0.0067	0.002	0.001	Shungin et al. (2015)
Height	450,112	−0.095	−0.0103	0.0017	2×10^{-9}	239,542	−0.0100	0.0033	0.0021	−0.010	0.0015	1×10^{-11}[a]	Wood et al. (2014)
Metabolic Trait													
ACR	437,029	0.003	0.0121	0.0021	6×10^{-9}	NA	NA	NA	NA	NA	NA	NA	NA
BP meds[c]	93,036/ 354,886	NA	0.0034	0.0008	0.000039	NA	NA	NA	NA	NA	NA	NA	NA
CAD[c]	37,741/ 318,892	NA	0.99	0.97–1.00	0.043	60,801/ 123,504[c]	1.000	0.98–1.02	0.99	NA	NA	NA	Nikpay et al. (2015)
DBP	449,332	0.13	0.0092	0.0020	0.0000038	111,783	0.111[d]	0.0460[d]	0.03	0.121[d]	0.0233[d]	2×10^{-7}[a]	Wain et al. (2017)
SBP	450,075	0.29	0.0120	0.0018	2×10^{-10}	108,620	0.157[d]	0.0734[d]	0.06	0.255[d]	0.0373[d]	9×10^{-12}[a]	Wain et al. (2017)
SBP[e]	378,880	0.34	0.0143	0.0024	2×10^{-9}	NA	NA	NA	NA	NA	NA	NA	NA
SBP adj.[f]	288,247	0.38	0.0159	0.0027	3×10^{-9}	NA	NA	NA	NA	NA	NA	NA	NA
SBP adj.[g]	284,360	0.37	0.0154	0.0027	1×10^{-8}	NA	NA	NA	NA	NA	NA	NA	NA
Hypertension[c]	241,691/ 206,525	NA	0.0051	0.0010	0.00000066	NA	NA	NA	NA	NA	NA	NA	NA
T2D[c]	14,371/ 428,017	NA	1.00	0.98–1.03	0.79	26,488/ 83,964[c]	1.01	0.97–1.04	0.72	1.006	0.99–1.03	0.51	Diabetes Genetics Replication and Meta-Analysis (DIAGRAM) Consortium et al. (2014)

UK Biobank data are based on 451,000 individuals of European ancestry corrected for relatedness. Effect sizes are in SDs after inverse normalization or ORs for disease traits. "Beta raw" refers to effect size in real units, centimeters, millimeters of mercury, and kilograms per square meter as applicable. None of the two sample meta-analyses showed evidence of heterogeneity at $p < 0.05$. ACR, albumin/creatine ratio; adj., adjusted; BP, blood pressure; CAD, coronary artery disease; circ., circumference; DBP, diastolic blood pressure; meds, medications; NA, data from variant not available in published GWAS; SBP, systolic blood pressure; T2D, type 2 diabetes.[a]Associations reaching $p < 0.0005$, a correction for the approximately 100 tests performed.

[b]Adjusted for height.

[c]Numbers of cases and controls.

[d]Effect sizes from Wain et al. (2017) in millimeters of mercury, p values genome corrected (GC).

[e]In subset of data, excluding related individuals.

[f]Adjusted for self-reported units alcohol per day. Adjusting for self-reported frequency of alcohol consumption (beta = 0.014, SE = 0.002, $p = 2 \times 10^{-9}$).

[g]Adjusted for self-reported units of alcohol per day, smoking, and salt intake.

Table 3. Associations between the Minor Allele at rs838133 and Liver, Lipid, and Glycemic Traits in Published GWAS

Trait	n	Beta	SE	p	GWAS Reference
ALP	30,846	−0.0043	0.0013	2.7×10^{-7}[a]	Chambers et al. (2011)
ALT	53,682	0.0028	0.0018	0.30	Chambers et al. (2011)
AST	39,020	0.0025	0.0022	0.25	Chambers et al. (2011)
GGT	55,885	0.0084	0.0023	3.7×10^{-6}[a]	Chambers et al. (2011)
HDL-C	92,820	0.0007	0.0055	0.96	Willer et al. (2013)
LDL-C	88,433	0.027	0.0059	1.7×10^{-5}[a]	Willer et al. (2013)
Triglycerides	89,485	0.0165	0.0052	0.002	Willer et al. (2013)
Fasting glucose	51,750	0.0021	0.0036	0.55	Scott et al. (2012)
Fasting insulin	51,750	−0.0004	0.0036	0.92	Scott et al. (2012)
HbA_{1c}	51,750	−0.0059	0.0042	0.15	Scott et al. (2012)
HOMAB	51,750	−0.0022	0.0037	0.55	Scott et al. (2012)
HOMAIR	51,750	−0.0022	0.0046	0.63	Scott et al. (2012)

Note that the variant is not present on the Metabochip genotyping array, meaning that sample sizes for these traits are appreciably smaller than those available in the UK Biobank. References for GWAS used are Chambers et al. (2011); Scott et al. (2012), and Willer et al. (2013). ALP, alkaline phosphatase; ALT, alanine aminotransferase; AST, aspartate aminotransferase; GGT, gamma-glutamyl transpeptidase; HDL-C, high-density lipoprotein cholesterol; HbA_{1c}, glycated hemoglobin; HOMAB, homeostatic model assessment of beta cell; HOMAIR, homeostatic model assessment of insulin resistance; LDL-C, low-density lipoprotein cholesterol.

[a]Associations reaching $p < 0.0005$, a correction for the approximately 100 tests performed.

in mice have identified links to growth, bone metabolism (Wei et al., 2012), circadian rhythms (Bookout et al., 2013), physical activity (Bookout et al., 2013), and reproductive traits (Owen et al., 2013). We therefore performed a "phenome-wide association study" (PheWAS), testing the association of the *FGF21* rs838133 variant with 82 traits in UK Biobank in the 451,000 individuals of European ancestry. We included several traits related to reward and risk behavior given the evidence that FGF21 affects reward function in the brain. We used a false discovery rate of 1% ($p < 0.0095$) to highlight associations (full list of 105 associations in Table S5). Notable associations, separate from the traits already mentioned, between the allele associated with higher sugar intake and other traits, included a more evening chronotype, lower activity (as measured by using the International Physical Activity Questionnaire [IPAQ]) and lower birth weight (all self-reported). There was no association with bone mineral density as measured by a heel ultrasound, but the sugar intake allele was associated with a nominally higher risk for osteoporosis (Table S5). There were no associations with the tested female reproductive traits. We further tested the associations with physical activity and sleep using a subset of 96,034 individuals who had worn accelerometer devices for 7 days and saw consistent associations, with nominal levels of statistical confidence (Table S6).

Phenotypes Associated with Other FGF21 Alleles Based on Publicly Available Data

Finally, we extended our analyses of human alleles likely to be affecting FGF21 function using publicly available data (http://www.type2diabetesgenetics.org). On the basis of whole-exome sequence data from 13,594 individuals, three protein truncating variants, each resulting in a frameshift, were present at amino acid positions S76, P171, and P178. These alleles were not associated with systolic blood pressure (SBP) or BMI (WHR was not available in the exome sequence data) but were present in only 7 individuals, and large effects could not be ruled out (e.g., up to 7.52 mm Hg for SBP and 6.94 kgm^2 for BMI).

DISCUSSION

Our findings are important for two main reasons. First, the genetic association data provide hypotheses about the potential adverse and beneficial effects of FGF21-based therapies. We used a common, naturally occurring variant in the human *FGF21* gene, rs838133, to generate these hypotheses. Second, the associations provide an advance in knowledge about the wide range of effects in humans of variation in the *FGF21* gene. Our motivation for studying the genotype-phenotype associations of the rs838133 variant came from the demonstration that the phenotypic associations of naturally occurring human genetic variation often mirror the effects of pharmacological manipulation of, or interventions targeting, the relevant protein or pathway linked to the gene. Examples include vitamin D and multiple sclerosis (Mokry et al., 2015), LDL cholesterol and coronary artery disease (Do et al., 2013), and LDL cholesterol and type 2 diabetes (Lotta et al., 2016). Although there is no direct evidence for the function of the rs838133 variant (or one in strong linkage disequilibrium), the A allele is very likely to represent lower FGF21 function, because it is very robustly associated with higher sugar and alcohol preference in people, a finding that is completely consistent with the genetic and pharmacological effects of FGF21 lowering in animal models, including non-human primates (von Holstein-Rathlou et al., 2016; Talukdar et al., 2016a). We note that there is no association between the *FGF21* rs838133 allele and *FGF21* expression in the liver, and it may be that the effects of this variant, or one in linkage disequilibrium, on *FGF21* gene expression are too subtle to pick up as an eQTL even in the relevant tissue.

Our data mean that researchers studying the molecular and therapeutic effects of FGF21 can now hypothesize and study in greater detail a number of potential mechanisms, in both experimental systems and clinical trials. These researchers can use the directions of effects associated with the rs838133 A allele we presented to generate hypotheses about the likely effects of lowering FGF21. Hypotheses about the likely effects of increasing FGF21 can be interpreted as the same effects but in opposite directions. The first hypothesis is that FGF21-based therapies will not have beneficial effects on type 2 diabetes. The lack of an association between the human *FGF21* allele and type 2 diabetes strengthens the inference for the lack of any anti-diabetic properties. Second, our data provide evidence for the hypothesis that FGF21 manipulation will have stronger effects on body-fat percentage and distribution than it will on absolute weight, as measured by BMI. The strength of the associations between the *FGF21* allele and higher WHR, despite associations with lower total body-fat percentage, were much stronger than any association with BMI. In contrast to the lack of association with type 2 diabetes, our data provide evidence for the putative effects of FGF21 on several clinically important metabolic traits, including blood pressure, ACR in the urine, and higher LDL cholesterol. None of these associations were previously known. The data also provide unequivocal statistical confidence that the rs838133 allele is associated with alcohol intake, compared with previous studies in which it was associated with alcohol intake with a p value of 0.03 (Soberg et al., 2017). In our analyses of UK Biobank and existing GWAS data, seven of these associations reached $p < 5 \times 10^{-5}$, and four reached conventional levels of genome-wide statistical confidence ($p < 5 \times 10^{-8}$).

The novel associations observed suggest that further functional and mechanistic studies are needed to investigate FGF21's role in adipocyte differentiation and storage capacity. An important observation for such studies is that our results suggest that FGF21's effects on body shape and blood pressure are unlikely to be secondary to the effect on macronutrient intake. These results suggest that FGF21 has pleiotropic effects, with separate effects on macronutrient intake to those on body shape and blood pressure. In contrast, there was a suggestion that the associations with lower total body-fat percentage were slightly attenuated when adjusting for macronutrient intake. These results are consistent with the minor *FGF21* allele's association with higher carbohydrate and lower protein and fat intake leading to slightly lower body-fat percentage.

The associations between body composition and blood pressure have similarities to those of the *Pro12Ala* allele in *PPARG*, in which the allele associated with lower total body-fat percentage is also associated with adverse metabolic effects, although there are clear effects of the *PPARG* allele with type 2 diabetes and insulin sensitivity in addition to body composition (Altshuler et al., 2000; Scott et al., 2012). Some studies have linked FGF21's function in adipocytes to those of PPARG (Wei et al., 2012), a transcription factor critical for adipogenesis and mutations that cause a form of lipodystrophy characterized by greatly reduced subcutaneous body fat, insulin resistance, high circulating triglyceride levels, and higher blood pressure (Savage et al., 2003). The *FGF21* rs838133 minor allele is not the first common allele to be associated with apparently paradoxical effects on fat mass and metabolic markers. Previous human genetic studies of alleles associated with insulin sensitivity have shown that most are also associated with an apparently paradoxical higher fat mass (and the insulin resistance allele is associated with lower fat mass) (Kilpelainen et al., 2011; Lotta et al., 2017; Scott et al., 2014; Shungin et al., 2015; Yaghootkar et al., 2014, 2016). For some of these alleles, the apparent paradox is explained by the higher fat mass being concentrated in the lower body, at least in women (e.g., those in or near *FAM*, *LYPLAL1*, and *GRB14*) (Shungin et al., 2015).

In addition to the anthropomorphic and metabolic associations, we observed some additional associations that will inform follow up experimental studies. On the basis of a PheWAS of 82 traits, we observed associations consistent with those observed in studies of mice that suggest FGF21 is a "global starvation" signal. For example, the allele that increases sugar intake was associated with lower levels of physical activity and altered chronotype, both features of mice with higher FGF21 (Bookout et al., 2013) but not with female reproductive traits or bone mineral density.

What are the limitations to using common human genetic variation to infer likely effects of therapies and experiments targeting a nearby gene? There are several limitations to our data. First, there is no direct experimental evidence that the rs838133 variant alters *FGF21* gene expression or function. We note that there was no association between the *FGF21* rs838133 allele and *FGF21* expression in the liver on the basis of 1,031 samples, and it may be that the effect of this variant, or one in linkage disequilibrium, on *FGF21* gene expression is too subtle to pick up as an eQTL, even in the relevant tissue. Despite occurring in an exon of *FGF21*, we cannot rule out the possibility that the variant operates through a nearby gene, and we note that the rs838133 allele is associated with the expression of a nearby gene *FUT2*, involved in vitamin B_{12} metabolism (Hazra et al., 2008). If the allele operates through a different gene, it would mean our inferences about any experimental or therapeutic interventions targeting FGF21 would be invalid. However, the fact that the human, mouse, and non-human primate nutrient preference phenotypes of FGF21 are identical suggests that this is unlikely. Second, the effects of the *FGF21* allele are extremely small, at approximately 0.33 mm Hg blood pressure and a 1 mm difference in hip circumference and height. However, the effect sizes of common genetic variants are not important when it comes to using them to provide insight into likely effects of much larger perturbations of a potential target. This point was recently illustrated by the very subtle but statistically robust effects of common alleles in genes encoding lipid-lowering proteins and type 2 diabetes (Ference et al., 2016; Lotta et al., 2016; Schmidt et al., 2017; Swerdlow and Sattar, 2015). Finally, the *FGF21* allele is very likely to influence human traits throughout life and therefore may have different effects compared with an acute, pharmacological, or experimental intervention.

In summary, human genetic association data provide further insight into the potential multiple metabolic effects of FGF21 and have hypothesis-generating implications for the development of therapies targeting FGF21.

EXPERIMENTAL PROCEDURES

UK Biobank Cohort

UK Biobank recruited more than 500,000 individuals aged 37–73 years (99.5% were between 40 and 69 years) between 2006 and 2010 from across the UK. Participants provided a range of information via questionnaires and interviews (e.g., demographics, health status, lifestyle), and anthropometric measurements, blood pressure readings, blood, urine, and saliva samples were taken for future analysis; this has been described in more detail elsewhere (Sudlow et al., 2015). SNP genotypes were generated from the Affymetrix Axiom UK Biobank array (~450,000 individuals) and the UK BiLEVE array (~50,000 individuals). This dataset underwent extensive central quality control (http://biobank.ctsu.ox.ac.uk). We based our study on 451,099 individuals of white European descent as defined by principal-component analysis (PCA). Briefly, principal components were generated in the 1000 Genomes cohort using high-confidence SNPs to obtain their individual loadings. These loadings were then used to project all of the UK Biobank samples into the same principal-component space, and individuals were then clustered using principal components 1–4. We removed 7 participants who withdrew from the study and 348 individuals whose self-reported sex did not match their genetic sex on the basis of relative intensities of X and Y chromosome SNP probe intensity.

FFQ and Alcohol Intake in UK Biobank Participants

The FFQ was added toward the end of the recruitment phase, and participants completed while at the recruitment center. Participants were then sent four FFQs and asked to complete them online. The questionnaire focused on the consumption of approximately 200 commonly consumed food and drinks (http://biobank.ctsu.ox.ac.uk/crystal/refer.cgi?id=118240). For each participant completing the FFQ, nutrient intakes were estimated by multiplying the quantity consumed by the nutrient composition of the food or beverage, as taken from the UK food composition database McCance and Widdowson's The Composition of Foods and its supplements. In total, 211,051 participants completed at least one FFQ. Participants were asked if this was a standard diet day for them, and we excluded the 18,054 participants who reported not following a standard diet. Averages were then calculated for participants with up to five normal questionnaires for 192,997 individuals.

We derived alcohol units consumed per day for individuals in the UK Biobank. For individuals reporting drinking alcohol at least once a week, a units-per-week variable was calculated, and for individuals reporting less frequent drinking, a units-per-month variable was calculated. A 125 mL glass of wine (red, white, or sparkling) was considered to be 1.5 units, a pint of beer or cider was considered to be 2.8 units, other alcoholic drinks (e.g., alcopops) were considered to be 1.5 units, and a measure of spirit was considered to be 1 unit.

Coffee, Smoking, and Salt Intake in UK Biobank Participants

All participants in the UK Biobank were asked about their smoking status, with individuals defined as never, former, or current smokers. All participants in the UK Biobank were asked about adding salt to food. Participants were asked, "Do you add salt to your food? (Do not include salt used in cooking)," with the options "never/rarely," "sometimes," "usually," and "always." In the general questionnaire, participants were asked, "How many cups of coffee do you drink each DAY? (Include decaffeinated coffee)." Participants were also asked about whether they drank caffeinated or decaffeinated coffee. From this we derived a number of cups of coffee per day and a number of cups of caffeinated coffee per day.

Disease and Related Anthropometric and Metabolic Traits in UK Biobank Participants

Measures of Adiposity

We used bio-impedance measures of body-fat percentage measured using the Tanita BC418MA body composition analyzer.

Measures of Disease and Disease-Related Traits

We defined type 2 diabetes, hypertension, blood pressure, and heart disease using baseline data and following similar definitions to those used in previous genome-wide association studies. For coronary artery disease, we additionally included cases from hospital episode statistics available at the time (through the March 31, 2016, release; International Classification of Diseases, Tenth Revision, codes I21*, I22*, I23*, I24*, and I25*). We defined type 2 diabetes cases if three criteria were present: (1) reports of either type 2 diabetes or generic diabetes at the interview, (2) at least a 1 year gap from diagnosis without requiring insulin, and (3) reported age at diagnosis over 35 years, to limit the number of individuals with slow-progressing autoimmune diabetes or monogenic forms. Individuals not reporting an age at diagnosis were excluded. We also excluded individuals diagnosed with diabetes within the year prior to the baseline study visit, as we were unable to determine whether they were using insulin within the first year. Controls were individuals not fulfilling these criteria.

We defined hypertensive patients as those with SBP of >140 mm Hg or diastolic blood pressure of >90 mm Hg or the report of blood pressure medication use. Controls were individuals not fulfilling these criteria. For the analysis of systolic and diastolic blood pressure, we corrected blood pressure measures in people on antihypertensive drugs by adding 15 mm Hg to systolic and 10 mm Hg to diastolic blood pressure, in keeping with the approach taken in genome-wide association studies.

We defined heart disease cases if individuals reported angina and/or a heart attack at the interview stage. We defined controls as individuals without these conditions.

Traits Derived from Accelerometers

The UK Biobank has collected accelerometer data from 103,711 participants, who wore the devices on the wrist for a continuous period of 1 week. We used a well-validated and freely available R package called GGIR (version 1.5-12) to process these files, made available to researchers, in order to extract measures of physical activity and sleep. For this study, we used three derived measures of activity and one measure of sleep timing (L5 time). L5 time represents the midpoint time of the least active 5 hr of the day, as defined by the minimum point of a moving average of activity levels. Our L5 time variable represents an individual's average across all days recorded, and units are reported in number of hours after previous midday. In addition, we also used measures of physical activity from the UK Biobank to define the proportion of activity, classified as (1) sedentary or asleep (<40 milligravities), (2) the proportion of activity at least non-sedentary (>40 milligravities), and (3) the proportion of activity over a threshold representing moderate to vigorous activity (>100 milligravities).

Traits in Published GWA Studies

We looked up the association of the variant rs838133 in existing relevant genome-wide association studies, as detailed in the tables. The variant was not present on the Metabochip.

Hepatic Expression Quantitative Trait Locus Analysis

Three hepatic eQTL datasets, comprising a total of 1,031 liver samples from individuals of European ancestry (Table S4), were analyzed in a meta-analysis (preliminary methods and results were reported by A.S. Etheridge et al., 2017, Am. Soc. Hum. Genet., conference). Tissue procurement, genotyping, and gene expression and eQTL analyses have been described previously for each of the three studies (Innocenti et al., 2011; Schadt et al., 2008; Greenawalt et al., 2011) Genotypes were imputed to the 1000 Genomes Project phase 1 reference panel with Minimac (https://genome.sph.umich.edu/wiki/Minimac), and expression probe sequences were mapped to Ensembl genes. Within each dataset, a genome-wide eQTL analysis was run with an additive genetic model including dataset-specific covariates to examine *cis* associations within a 100 kb flanking window. Results from the three datasets were then combined with a modified meta-test statistic that was calculated using the following approach: $t_{meta} = (\sum w_i t_i)/\sqrt{(\sum w_i^2)}$, $w = \sqrt{(n-(\#covariates)-1)}$ where i is datasets 1–3 and *n* is sample size. Generation of p values was accomplished by assuming that the meta-test statistics were normally distributed.

Statistical Analysis

All genotype-phenotype association data were generated starting from 451,099 individuals defined as European ancestry and using BOLT-LMM

version 1.2, which uses an LD score regression approach to account for structure caused by relatedness (close and distant). All association testing was based on an additive, per allele model and adjusted for SNP chip type (UKB Axiom or UK BiLEVE), test center, sex, and age (or year of birth for age at menarche). Accelerometry-based phenotypes were additionally adjusted for season and age at wear time. We tested approximately 100 traits and so highlight main associations reaching $p < 0.0005$, but given that several metabolic and anthropometric traits reach genome-wide significance, and the known role of the *FGF21* variant, we mention other traits reaching nominal significance and used a false discovery rate of 1% to highlight associations in the PheWAS. For continuous traits, we inverse normalized phenotypes to account for any skewed distributions.

SUPPLEMENTAL INFORMATION

Supplemental Information includes six tables and can be found with this article online at https://doi.org/10.1016/j.celrep.2018.03.070.

ACKNOWLEDGMENTS

A.R.W. and T.M.F. are supported by the European Research Council (grant 323195:GLUCOSEGENES-FP7-IDEAS-ERC). R.M.F. is a Sir Henry Dale Fellow (Wellcome Trust and Royal Society grant 104150/Z/14/Z). H.Y. is an RD Lawrence Fellow, funded by Diabetes UK. R.N.B. is funded by the Wellcome Trust and Royal Society grant 104150/Z/14/Z. J.T. is funded by the European Regional Development Fund (ERDF) and a Diabetes Research and Wellness Foundation fellowship. S.E.J. is funded by the Medical Research Council (grant MR/M005070/1). M.A.T., M.N.W., and A.M. are supported by the Wellcome Trust Institutional Strategic Support Award (WT097835MF). C.M.L. is supported by the Li Ka Shing Foundation, by the National Institute for Health Research (NIHR) Biomedical Research Centre (Oxford), by Widenlife, and by the NIH (CRR00070 CR00.01). The funders had no influence on study design, data collection and analysis, decision to publish, or preparation of the manuscript. The Novo Nordisk Foundation Center for Basic Metabolic Research is an independent research center at the University of Copenhagen partially funded by an unrestricted donation from the Novo Nordisk Foundation (http://cbmr.ku.dk). This research has been conducted using the UK Biobank resource. This work was carried out under UK Biobank project numbers 9072 and 9055. We thank Alexei Kharitonenkov, Robert Andrews, and Tim McDonald for helpful comments. Details of patient and public involvement in the UK Biobank are available online (http://www.ukbiobank.ac.uk/about-biobank-uk/ and https://www.ukbiobank.ac.uk/wp-content/uploads/2011/07/Summary-EGF-consultation.pdf?phpMyAdmin=trmKQlYdjjnQIgJ%2CfAzikMhEnx6). No patients were specifically involved in setting the research question or the outcome measures, nor were they involved in developing plans for recruitment, design, or implementation of this study. No patients were asked to advise on interpretation or writing up of results. There are no specific plans to disseminate the results of the research to study participants, but the UK Biobank disseminates key findings from projects on its website.

AUTHOR CONTRIBUTIONS

T.M.F. designed and led the study and wrote the manuscript. R.N.B., S.E.J., H.Y., M.A.T., K.S.R., F.C., S.S., Y.J., W.T., and J.H. curated, quality-controlled, and analyzed the UK Biobank data. B.W. performed PheWAS. J.L. conducted bioinformatics analysis of variants' properties. A.S.E., P.J.G., D.J., F.W., Y.Z., and F.I. provided liver eQTL data. C.M.L. provided expertise on body-fat distribution phenotypes. N.G. provided expertise on FGF21 variant and human phenotypes. A.M., R.M.F., and M.N.W. supervised analyses of UK Biobank data. J.T. and A.R.W. led key analytical pipelines for analysis of UK Biobank data.

DECLARATION OF INTERESTS

The authors declare no competing interests.

Received: October 15, 2017
Revised: December 19, 2017
Accepted: March 15, 2018
Published: April 10, 2018

REFERENCES

Altshuler, D., Hirschhorn, J.N., Klannemark, M., Lindgren, C.M., Vohl, M.C., Nemesh, J., Lane, C.R., Schaffner, S.F., Bolk, S., Brewer, C., et al. (2000). The common PPARgamma Pro12Ala polymorphism is associated with decreased risk of type 2 diabetes. Nat. Genet. *26*, 76–80.

Berglund, E.D., Li, C.Y., Bina, H.A., Lynes, S.E., Michael, M.D., Shanafelt, A.B., Kharitonenkov, A., and Wasserman, D.H. (2009). Fibroblast growth factor 21 controls glycemia via regulation of hepatic glucose flux and insulin sensitivity. Endocrinology *150*, 4084–4093.

Bondurant, L.D., Ameka, M., Naber, M.C., Markan, K.R., Idiga, S.O., Acevedo, M.R., Walsh, S.A., Ornitz, D.M., and Potthoff, M.J. (2017). FGF21 regulates metabolism through adipose-dependent and -independent mechanisms. Cell Metab. *25*, 935–944.e4.

Bookout, A.L., de Groot, M.H., Owen, B.M., Lee, S., Gautron, L., Lawrence, H.L., Ding, X., Elmquist, J.K., Takahashi, J.S., Mangelsdorf, D.J., and Kliewer, S.A. (2013). FGF21 regulates metabolism and circadian behavior by acting on the nervous system. Nat. Med. *19*, 1147–1152.

Chambers, J.C., Zhang, W., Sehmi, J., Li, X., Wass, M.N., Van Der Harst, P., Holm, H., Sanna, S., Kavousi, M., Baumeister, S.E., et al. (2011). Genome-wide association study identifies loci influencing concentrations of liver enzymes in plasma. Nat. Genet. *43*, 1131–1138.

Chu, A.Y., Workalemahu, T., Paynter, N.P., Rose, L.M., Giulianini, F., Tanaka, T., Ngwa, J.S., Qi, Q., Curhan, G.C., Rimm, E.B., et al.; CHARGE Nutrition Working Group; DietGen Consortium (2013). Novel locus including FGF21 is associated with dietary macronutrient intake. Hum. Mol. Genet. *22*, 1895–1902.

Coskun, T., Bina, H.A., Schneider, M.A., Dunbar, J.D., Hu, C.C., Chen, Y., Moller, D.E., and Kharitonenkov, A. (2008). Fibroblast growth factor 21 corrects obesity in mice. Endocrinology *149*, 6018–6027.

Diabetes Genetics Replication and Meta-Analysis (DIAGRAM) Consortium; Asian Genetic Epidemiology Network Type 2 Diabetes (AGEN-T2D) Consortium; South Asian Type 2 Diabetes (SAT2D) Consortium; Mexican American Type 2 Diabetes (MAT2D) Consortium; Type 2 Diabetes Genetic Exploration by Next-Generation Sequencing in Multi-Ethnic Samples (T2D-GENES) Consortium; Mahajan, A., Go, M.J., Zhang, W., Below, J.E., Gaulton, K.J., et al. (2014). Genome-wide trans-ancestry meta-analysis provides insight into the genetic architecture of type 2 diabetes susceptibility. Nat. Genet. *46*, 234–244.

Do, R., Willer, C.J., Schmidt, E.M., Sengupta, S., Gao, C., Peloso, G.M., Gustafsson, S., Kanoni, S., Ganna, A., Chen, J., et al. (2013). Common variants associated with plasma triglycerides and risk for coronary artery disease. Nat. Genet. *45*, 1345–1352.

Ference, B.A., Robinson, J.G., Brook, R.D., Catapano, A.L., Chapman, M.J., Neff, D.R., Voros, S., Giugliano, R.P., Davey Smith, G., Fazio, S., and Sabatine, M.S. (2016). Variation in PCSK9 and HMGCR and risk of cardiovascular disease and diabetes. N. Engl. J. Med. *375*, 2144–2153.

Frayling, T.M. (2015). Statins and type 2 diabetes: genetic studies on target. Lancet *385*, 310–312.

Gaich, G., Chien, J.Y., Fu, H., Glass, L.C., Deeg, M.A., Holland, W.L., Kharitonenkov, A., Bumol, T., Schilske, H.K., and Moller, D.E. (2013). The effects of LY2405319, an FGF21 analog, in obese human subjects with type 2 diabetes. Cell Metab. *18*, 333–340.

Greenawalt, D.M., Dobrin, R., Chudin, E., Hatoum, I.J., Suver, C., Beaulaurier, J., Zhang, B., Castro, V., Zhu, J., Sieberts, S.K., et al. (2011). A survey of the genetics of stomach, liver, and adipose gene expression from a morbidly obese cohort. Genome Res. *21*, 1008–1016.

Hazra, A., Kraft, P., Selhub, J., Giovannucci, E.L., Thomas, G., Hoover, R.N., Chanock, S.J., and Hunter, D.J. (2008). Common variants of FUT2 are associated with plasma vitamin B12 levels. Nat. Genet. *40*, 1160–1162.

Hurle, M.R., Nelson, M.R., Agarwal, P., and Cardon, L.R. (2016). Impact of genetically supported target selection on R&D productivity. Nat. Rev. Drug Discov. Published online September 12, 2016. https://doi.org/10.1038/nrd.2016.187.

Innocenti, F., Cooper, G.M., Stanaway, I.B., Gamazon, E.R., Smith, J.D., Mirkov, S., Ramirez, J., Liu, W., Lin, Y.S., Moloney, C., et al. (2011). Identification, replication, and functional fine-mapping of expression quantitative trait loci in primary human liver tissue. PLoS Genet. *7*, e1002078.

Kharitonenkov, A., and DiMarchi, R. (2017). Fibroblast growth factor 21 night watch: advances and uncertainties in the field. J. Intern. Med. *281*, 233–246.

Kharitonenkov, A., Shiyanova, T.L., Koester, A., Ford, A.M., Micanovic, R., Galbreath, E.J., Sandusky, G.E., Hammond, L.J., Moyers, J.S., Owens, R.A., et al. (2005). FGF-21 as a novel metabolic regulator. J. Clin. Invest. *115*, 1627–1635.

Kharitonenkov, A., Wroblewski, V.J., Koester, A., Chen, Y.F., Clutinger, C.K., Tigno, X.T., Hansen, B.C., Shanafelt, A.B., and Etgen, G.J. (2007). The metabolic state of diabetic monkeys is regulated by fibroblast growth factor-21. Endocrinology *148*, 774–781.

Kilpelainen, T.O., Zillikens, M.C., Stancakova, A., Finucane, F.M., Ried, J.S., Langenberg, C., Zhang, W., Beckmann, J.S., Luan, J., Vandenput, L., et al. (2011). Genetic variation near IRS1 associates with reduced adiposity and an impaired metabolic profile. Nat. Genet. *43*, 753–760.

Kim, A.M., Somayaji, V.R., Dong, J.Q., Rolph, T.P., Weng, Y., Chabot, J.R., Gropp, K.E., Talukdar, S., and Calle, R.A. (2017). Once-weekly administration of a long-acting fibroblast growth factor 21 analogue modulates lipids, bone turnover markers, blood pressure and body weight differently in obese people with hypertriglyceridaemia and in non-human primates. Diabetes Obes. Metab. *19*, 1762–1772.

Locke, A.E., Kahali, B., Berndt, S.I., Justice, A.E., Pers, T.H., Day, F.R., Powell, C., Vedantam, S., Buchkovich, M.L., Yang, J., et al. (2015). Genetic studies of body mass index yield new insights for obesity biology. Nature *518*, 197–206.

Lotta, L.A., Sharp, S.J., Burgess, S., Perry, J.R.B., Stewart, I.D., Willems, S.M., Luan, J., Ardanaz, E., Arriola, L., Balkau, B., et al. (2016). Association between low-density lipoprotein cholesterol-lowering genetic variants and risk of type 2 diabetes: a meta-analysis. JAMA *316*, 1383–1391.

Lotta, L.A., Gulati, P., Day, F.R., Payne, F., Ongen, H., van de Bunt, M., Gaulton, K.J., Eicher, J.D., Sharp, S.J., Luan, J., et al.; EPIC-InterAct Consortium; Cambridge FPLD1 Consortium (2017). Integrative genomic analysis implicates limited peripheral adipose storage capacity in the pathogenesis of human insulin resistance. Nat. Genet. *49*, 17–26.

Lu, Y., Day, F.R., Gustafsson, S., Buchkovich, M.L., Na, J., Bataille, V., Cousminer, D.L., Dastani, Z., Drong, A.W., Esko, T., et al. (2016). New loci for body fat percentage reveal link between adiposity and cardiometabolic disease risk. Nat. Commun. *7*, 10495.

Mokry, L.E., Ross, S., Ahmad, O.S., Forgetta, V., Smith, G.D., Goltzman, D., Leong, A., Greenwood, C.M., Thanassoulis, G., and Richards, J.B. (2015). Vitamin D and risk of multiple sclerosis: a Mendelian randomization study. PLoS Med. *12*, e1001866.

Nikpay, M., Goel, A., Won, H.H., Hall, L.M., Willenborg, C., Kanoni, S., Saleheen, D., Kyriakou, T., Nelson, C.P., Hopewell, J.C., et al. (2015). A comprehensive 1,000 Genomes-based genome-wide association meta-analysis of coronary artery disease. Nat. Genet. *47*, 1121–1130.

Owen, B.M., Bookout, A.L., Ding, X., Lin, V.Y., Atkin, S.D., Gautron, L., Kliewer, S.A., and Mangelsdorf, D.J. (2013). FGF21 contributes to neuroendocrine control of female reproduction. Nat. Med. *19*, 1153–1156.

Reitman, M.L. (2013). FGF21 mimetic shows therapeutic promise. Cell Metab. *18*, 307–309.

Savage, D.B., Tan, G.D., Acerini, C.L., Jebb, S.A., Agostini, M., Gurnell, M., Williams, R.L., Umpleby, A.M., Thomas, E.L., Bell, J.D., et al. (2003). Human metabolic syndrome resulting from dominant-negative mutations in the nuclear receptor peroxisome proliferator-activated receptor-gamma. Diabetes *52*, 910–917.

Schadt, E.E., Molony, C., Chudin, E., Hao, K., Yang, X., Lum, P.Y., Kasarskis, A., Zhang, B., Wang, S., Suver, C., et al. (2008). Mapping the genetic architecture of gene expression in human liver. PLoS Biol. *6*, e107.

Schmidt, A.F., Swerdlow, D.I., Holmes, M.V., Patel, R.S., Fairhurst-Hunter, Z., Lyall, D.M., Hartwig, F.P., Horta, B.L., Hypponen, E., Power, C., et al. (2017). PCSK9 genetic variants and risk of type 2 diabetes: a Mendelian randomisation study. Lancet Diabetes Endocrinol. *5*, 97–105.

Scott, R.A., Fall, T., Pasko, D., Barker, A., Sharp, S.J., Arriola, L., Balkau, B., Barricarte, A., Barroso, I., Boeing, H., et al.; RISC study group; EPIC-InterAct Consortium (2014). Common genetic variants highlight the role of insulin resistance and body fat distribution in type 2 diabetes, independent of obesity. Diabetes *63*, 4378–4387.

Scott, R.A., Lagou, V., Welch, R.P., Wheeler, E., Montasser, M.E., Luan, J., Magi, R., Strawbridge, R.J., Rehnberg, E., Gustafsson, S., et al. (2012). Large-scale association analyses identify new loci influencing glycemic traits and provide insight into the underlying biological pathways. Nat. Genet. *44*, 991–1005.

Shungin, D., Winkler, T.W., Croteau-Chonka, D.C., Ferreira, T., Locke, A.E., Magi, R., Strawbridge, R.J., Pers, T.H., Fischer, K., Justice, A.E., et al. (2015). New genetic loci link adipose and insulin biology to body fat distribution. Nature *518*, 187–196.

Soberg, S., Sandholt, C.H., Jespersen, N.Z., Toft, U., Madsen, A.L., Von Holstein-Rathlou, S., Grevengoed, T.J., Christensen, K.B., Bredie, W.L.P., Potthoff, M.J., et al. (2017). FGF21 is a sugar-induced hormone associated with sweet intake and preference in humans. Cell Metab. *25*, 1045–1053.e6.

Sudlow, C., Gallacher, J., Allen, N., Beral, V., Burton, P., Danesh, J., Downey, P., Elliott, P., Green, J., Landray, M., et al. (2015). UK Biobank: an open access resource for identifying the causes of a wide range of complex diseases of middle and old age. PLoS Med. *12*, e1001779.

Swerdlow, D.I., and Sattar, N. (2015). Blood lipids and type 2 diabetes risk: can genetics help untangle the web? Diabetes *64*, 2344–2345.

Swerdlow, D.I., Preiss, D., Kuchenbaecker, K.B., Holmes, M.V., Engmann, J.E., Shah, T., Sofat, R., Stender, S., Johnson, P.C., Scott, R.A., et al. (2015). HMG-coenzyme A reductase inhibition, type 2 diabetes, and bodyweight: evidence from genetic analysis and randomised trials. Lancet *385*, 351–361.

Talukdar, S., Owen, B.M., Song, P., Hernandez, G., Zhang, Y., Zhou, Y., Scott, W.T., Paratala, B., Turner, T., Smith, A., et al. (2016a). FGF21 regulates sweet and alcohol preference. Cell Metab. *23*, 344–349.

Talukdar, S., Zhou, Y., Li, D., Rossulek, M., Dong, J., Somayaji, V., Weng, Y., Clark, R., Lanba, A., Owen, B.M., et al. (2016b). A long-acting FGF21 molecule, PF-05231023, decreases body weight and improves lipid profile in non-human primates and type 2 diabetic subjects. Cell Metab. *23*, 427–440.

Tanaka, T., Ngwa, J.S., van Rooij, F.J., Zillikens, M.C., Wojczynski, M.K., Frazier-Wood, A.C., Houston, D.K., Kanoni, S., Lemaitre, R.N., Luan, J., et al. (2013). Genome-wide meta-analysis of observational studies shows common genetic variants associated with macronutrient intake. Am. J. Clin. Nutr. *97*, 1395–1402.

von Holstein-Rathlou, S., BonDurant, L.D., Peltekian, L., Naber, M.C., Yin, T.C., Claflin, K.E., Urizar, A.I., Madsen, A.N., Ratner, C., Holst, B., et al. (2016). FGF21 mediates endocrine control of simple sugar intake and sweet taste preference by the liver. Cell Metab. *23*, 335–343.

Wain, L.V., Vaez, A., Jansen, R., Joehanes, R., Van Der Most, P.J., Erzurumluoglu, A.M., O'Reilly, P.F., Cabrera, C.P., Warren, H.R., Rose, L.M., et al. (2017). Novel blood pressure locus and gene discovery using genome-wide association study and expression data sets from blood and the kidney. Hypertension, Published online July 24, 2017. https://doi.org/10.1161/HYPERTENSIONAHA.117.09438.

Wei, W., Dutchak, P.A., Wang, X., Ding, X., Wang, X., Bookout, A.L., Goetz, R., Mohammadi, M., Gerard, R.D., Dechow, P.C., et al. (2012). Fibroblast growth

factor 21 promotes bone loss by potentiating the effects of peroxisome proliferator-activated receptor γ. Proc. Natl. Acad. Sci. USA *109*, 3143–3148.

Willer, C.J., Schmidt, E.M., Sengupta, S., Peloso, G.M., Gustafsson, S., Kanoni, S., Ganna, A., Chen, J., Buchkovich, M.L., Mora, S., et al. (2013). Discovery and refinement of loci associated with lipid levels. Nat. Genet. *45*, 1274–1283.

Wood, A.R., Esko, T., Yang, J., Vedantam, S., Pers, T.H., Gustafsson, S., Chu, A.Y., Estrada, K., Luan, J., Kutalik, Z., et al. (2014). Defining the role of common variation in the genomic and biological architecture of adult human height. Nat. Genet. *46*, 1173–1186.

Yaghootkar, H., Scott, R.A., White, C.C., Zhang, W., Speliotes, E., Munroe, P.B., Ehret, G.B., Bis, J.C., Fox, C.S., Walker, M., et al. (2014). Genetic evidence for a normal-weight “metabolically obese” phenotype linking insulin resistance, hypertension, coronary artery disease, and type 2 diabetes. Diabetes *63*, 4369–4377.

Yaghootkar, H., Lotta, L.A., Tyrrell, J., Smit, R.A., Jones, S.E., Donnelly, L., Beaumont, R., Campbell, A., Tuke, M.A., Hayward, C., et al. (2016). Genetic evidence for a link between favorable adiposity and lower risk of type 2 diabetes, hypertension, and heart disease. Diabetes *65*, 2448–2460.

Cell Reports, Volume *23*

Supplemental Information

A Common Allele in FGF21 Associated with Sugar Intake Is Associated with Body Shape, Lower Total Body-Fat Percentage, and Higher Blood Pressure

Timothy M. Frayling, Robin N. Beaumont, Samuel E. Jones, Hanieh Yaghootkar, Marcus A. Tuke, Katherine S. Ruth, Francesco Casanova, Ben West, Jonathan Locke, Seth Sharp, Yingjie Ji, William Thompson, Jamie Harrison, Amy S. Etheridge, Paul J. Gallins, Dereje Jima, Fred Wright, Yihui Zhou, Federico Innocenti, Cecilia M. Lindgren, Niels Grarup, Anna Murray, Rachel M. Freathy, Michael N. Weedon, Jessica Tyrrell, and Andrew R. Wood

SUPPLEMENTARY INFORMATION

Supplementary table 1. Details of UK Biobank participants taking Food frequency questionnaire, related to Table 1.

Demographic	Completed FFQ	Not completed FFQ	P		Demographic	Completed FFQ more than once	Completed FFQ once only	P
N	188,060	120,185			N	117,624	70,436	
Age at recruitment (SD)	56.8 (7.8)	56.3 (8.1)	$<1x10^{-15}$		Age at recruitment (SD)	56.8 (7.8)	56.8 (8.0)	0.58
Male, N(%)	84,976 (45.2)	58,128 (48.4)	$<1x10^{-15}$		Male, N(%)	52,298 (44.5)	32,678 (46.4)	$<1x10^{-15}$
Mean BMI (SD)	26.9 (4.6)	27.6 (4.7)	$<1x10^{-15}$		Mean BMI (SD)	26.7 (4.6)	27.2 (4.7)	$<1x10^{-15}$
Mean body fat percentage (SD)	30.8 (8.4)	31.3 (8.4)	$<1x10^{-15}$		Mean body fat percentage (SD)	30.6 (8.4)	31.1 (8.4)	$<1x10^{-15}$
Mean systolic blood pressure (SD)	142 (23)	143 (24)	$<1x10^{-15}$		Mean systolic blood pressure (SD)	142 (23)	143 (24)	$2x10^{-8}$
Mean waist circumference (SD)	89.0 (13.2)	90.9 (13.4)	$<1x10^{-15}$		Mean waist circumference (SD)	88.6 (13.3)	89.9 (13.4)	$<1x10^{-15}$
Mean hip circumference (SD)	103 (9)	104 (9)	$<1x10^{-15}$		Mean hip circumference (SD)	103 (9)	103 (9)	$<1x10^{-15}$
Mean WHR (SD)	0.86 (0.09)	0.87 (0.09)	$<1x10^{-15}$		Mean WHR (SD)	0.86 (0.09)	0.87 (0.09)	$<1x10^{-15}$
Mean ACR (SD)	1.62 (2.2)	1.57 (2.4)	0.001		Mean ACR (SD)	1.63 (2.21)	1.61 (2.35)	0.56
T2D, N (%)	5,045 (2.7)	3,590 (3.0)	$2x10^{-12}$		T2D, N (%)	2,978 (2.5)	2,067 (2.9)	$3x10^{-6}$
CAD, N(%)	12,262 (6.5)	9,452 (7.9)	$<1x10^{-15}$		CAD, N(%)	7,066 (6.0)	5,196 (7.4)	$<1x10^{-15}$

Supplementary table 2. Details of the association between rs838133 and anthropometric traits in men and women separately, related to Table 2.

Anthropometric trait	Sex	BETA	SE	P
Body fat percentage	men	-0.00750807	0.003019	0.012
Body fat percentage	women	-0.00950441	0.002766	0.0012
BMI	men	-0.00443286	0.003055	0.11
BMI	women	-0.00582809	0.0028	0.056
Hip circumference	men	-0.0134019	0.003065	8.9E-06
Hip circumference	women	-0.0135767	0.002797	2.1E-06
Waist circumference	men	-0.00466646	0.003066	0.082
Waist circumference	women	-0.00519281	0.002795	0.063
WHRadjBMI	men	0.0114921	0.003166	0.00022
WHRadjBMI	women	0.00632038	0.002825	0.048
WHR	men	0.00575562	0.003136	0.061
WHR	women	0.00991502	0.002817	0.0004
Height	men	-0.0105833	0.002691	0.00053
Height	women	-0.0125656	0.002426	5.4E-08

Supplementary table 3. Associations between the minor A allele at rs838133 and anthropometric and metabolic traits in a subset of individuals with food frequency questionnaire data available, before and after adjusting for percentage macronutrient intake, related to Table 2.

Adjusting for percentage carbs, fat and protein					Unadjusted in same number		
**Trait**	**N**	**Beta SD or OR**	**SE or 95%CI**	**P**	**Beta SD or OR**	**SE or 95%CI**	**P**
Body fat %	148609	-0.0049	0.0038	0.19	-0.0088	0.0038	0.021
BMI	150514	-0.0042721	0.0037	0.25	-0.0084	0.0037	0.025
Hip circumference	150792	-0.0093	0.0037	0.012	-0.0129	0.0037	$5x10^{-4}$
Waist circumference	150804	-0.0024	0.0037	0.51	-0.0059	0.0037	0.12
WHRadjBMI	150483	0.0086	0.0037	0.021	0.0089	0.0037	0.017
WHR	150778	0.0053	0.0037	0.16	0.0035	0.0038	0.35
Height	150761	-0.0082	0.0037	0.029	-0.0077	0.0037	0.04
ACR	146872	0.0074	0.0038	0.049	0.0083	0.0038	0.027
BP meds**	27453/122939	1.02	1.00-1.04	0.039	1.02	1.00-1.04	0.07
CAD**	9871/104533	1	0.97-1.03	0.92	1	0.97-1.03	0.88
DBP	150477	0.0113	0.0037	0.002	0.0108	0.0037	0.004
SBP	150690	0.0126	0.0037	0.0007	0.0125	0.0037	0.0007
Hypertension**	75611/74683	1.03	1.01-1.04	0.0007	1.03	1.01-1.04	0.0009
T2D**	4079/144599	0.96	0.92-1.01	0.11	0.95	0.91-1.00	0.036

Supplementary Table 4. Association between rs439523, in LD with rs838133 ($r^2 = 0.62$), and *FGF21* expression in human liver. Within three liver eQTL data sets, linear regression was used to model *FGF21* expression levels with adjustment for relevant covariates. Results from the three liver datasets were combined by meta-analysis. *FGF21* expression level was determined using microarray and only included patients of European ancestry. The data was coded such that a negative beta means that as the number of minor alleles increases there is a decrease in *FGF21* expression, related to experimental procedures.

				rs439523		
**Dataset**	**n**	**Expression**	**Genotyping**	**P-value**	**Beta**	**PMID**
Dataset 1	164	Agilent-014850 Whole Human Genome 4x44K gene expression (NCBI GEO accession: GSE25935)	Illumina Human610-Quad v1.0 BeadChip (NCBI GEO accession: GSE26105)	0.0811	-0.0141	21637794
Dataset 2	286	Agilent Technologies (NCBI GEO accession: GSE9588)	Affymetrix GeneChip Human Mapping 500k genotyping microarray	0.4700	-0.0055	18462017
Dataset 3	581	Agilent Technologies (NCBI GEO accession: GSE9588)	HumanHap 650Y	0.9643	-0.0002	21602305
Meta	1031			0.3076	$t_{meta} = -1.0202$	

Supplementary Table 5. "Phenome wide association study" in 451,099 individuals. Associations between the rs838133 variant and 82 traits in UK Biobank. Emboldened rows highlight associations reaching false discovery rates of 1% or less (p<0.012), related to experimental procedures.

Trait	BETA	SE	P
**Systolic blood pressure**	***0.0120336***	***0.00184876***	***2E-10***
**Hip circumference**	***-0.0121696***	***0.00203943***	***8E-10***
**Height**	***-0.0103052***	***0.00171557***	***2.4E-09***
**Albumin creatine ratio, continous**	***0.0121483***	***0.00208866***	***6.2E-09***
**Hypertension**	***0.00506564***	***0.000988123***	***0.00000066***
**Fat free mass in the arm (bioimpedence)**	***-0.00564884***	***0.00124359***	***0.0000031***
**Diastolic blood pressure**	***0.00920308***	***0.001977***	***0.0000038***
**Body fat mass (bioimpedence)**	***-0.00907592***	***0.00197836***	***0.0000039***
**Waist hip ratio adjusted for BMI**	***0.0100678***	***0.0022142***	***0.000004***
**Blood pressure medication**	***0.00342205***	***0.00083121***	***0.000039***
**Body fat mass % (bioimpedence)**	***-0.00602927***	***0.0015396***	***0.00013***
**Body fat free mass % (bioimpedence)**	***-0.00428308***	***0.00122452***	***0.00024***
**Chronotype (morning or evening person)**	***-0.00987515***	***0.00278196***	***0.00058***
**IPAQ activity**	***-0.00728667***	***0.0022639***	***0.0013***
**Waist hip ratio**	***0.00676474***	***0.00220517***	***0.0022***
**Father's age at death**	***-0.00750113***	***0.00252796***	***0.003***
**Birth weight**	***-0.00855478***	***0.00283002***	***0.0032***
**Lost weight in the last year**	***-0.00312441***	***0.00105482***	***0.0032***
**Limb fat mass (bioimpedence)**	***-0.00379891***	***0.00134292***	***0.0061***
**Psoriasis**	***0.000840724***	***0.000334925***	***0.0091***
**Osteoporosis**	***0.000717142***	***0.000280175***	***0.0095***
**Age first child born**	***0.00802482***	***0.00312645***	***0.0096***
Albumin creatinine ratio using cut off of <=3.5	*0.00150966*	*0.000608407*	*0.014*
diverticular disease ICD10	*0.00121587*	*0.000534468*	*0.02*
Waist circumference	*-0.00391468*	*0.00180551*	*0.022*
Number of children fathered	*-0.00702426*	*0.00317092*	*0.027*
Length of menstrual cycle	*0.0167682*	*0.00771689*	*0.039*
Age at menarche	*-0.00906782*	*0.00447201*	*0.042*
Coronary artery disease	*0.00145283*	*0.000728024*	*0.043*
undersleeper	0.00201585	0.000991189	0.046
Ectopic pregnancy	-0.000234989	0.000119476	0.049
Major Depresssion	-0.000304361	0.000164135	0.064
Body mass index	-0.00349568	0.00213527	0.1
Ovarian cysts	0.000603848	0.000367178	0.1
Fibroids	0.000736002	0.000497518	0.14
Arterial Stiffness	-0.00526339	0.00363522	0.16
Number of births	-0.00413257	0.00287968	0.16
Major depressive disorder	0.000998134	0.00073579	0.17
Low grip strength in >=60 year olds	0.00143402	0.00113557	0.2
Number of pregnancies - women	-0.00341784	0.00294489	0.25

Trait	BETA	SE	P
Napping	0.00142163	0.00125651	0.25
Infertile	0.00015435	0.000143199	0.28
Mother's age at death	-0.00300551	0.00279656	0.28
Migraine	-0.000394451	0.000378319	0.29
Still birth	0.000483813	0.000455413	0.29
First child preterm?	0.000711064	0.000695798	0.31
preterm birth	-0.000597964	0.000604261	0.32
First child's birthweight	-0.00302311	0.00331566	0.34
Myocardial infarction	0.000288971	0.000325801	0.38
Major depressive disorder - recurrent	0.000572699	0.000674668	0.4
Reproductive life span	-0.0029088	0.00427444	0.45
Hours slept (excluding <3, >14)	-0.00168035	0.00233723	0.48
Major depressive disorder - single episode	0.000278265	0.000403138	0.49
Polycystic ovarian syndrome	-9.72881E-05	0.000139946	0.49
Fried index: 0-2 (not frail) 3-5 (frail)	-0.000420167	0.000659628	0.52
Spontaneous miscarriage	0.000751262	0.00120625	0.53
Gastro-oesophageal reflux syndrome	-0.00046589	0.000763007	0.55
Gestational diabetes	-0.000140713	0.000237492	0.55
Oversleeper	0.000389081	0.000751036	0.59
Have at least one of gord, h_hernia, Barrett's c	-0.000447127	0.000864513	0.6
Osteoarthritis	-0.000301594	0.000752518	0.71
Breast Cancer	0.000305281	0.000659173	0.71
Ovarian cancer	7.19151E-05	0.000206199	0.73
Menopause before age 45, age >50 as controls	-0.000211173	0.00133303	0.75
endometriosis	0.000120854	0.000365912	0.75
number of pregnancies lost	-0.000926449	0.00297796	0.76
Cancer registry yes no	0.000262795	0.000727278	0.77
Insomnia (3 categories)	0.000421846	0.00153804	0.78
Age at menopause < 40 years	-0.000089953	0.000338761	0.79
sedentary time	-0.000583996	0.00209313	0.79
Type 2 diabetes strict defintion	9.62441E-05	0.000385218	0.79
Snorer	-0.000241752	0.00104514	0.8
number of still births	-0.000587357	0.00297666	0.84
Gain weight in the last year	-0.000237803	0.00110528	0.85
Premature ovarian insufficiency (Cases are nat	4.00769E-06	0.00168513	0.85
number of miscarriages	0.000505664	0.00297698	0.87
Uterine cancer	3.04602E-05	0.000241324	0.9
Natural age at menopause	0.000414949	0.00432607	0.95
Bone mineral density	-0.000632625	0.00190941	0.96
preeclampsia	-1.04041E-05	0.000275402	0.97
Menorrhagia	2.36807E-06	0.000229386	0.99

Supplementary table 6. Validation of associations of physical activity and chronotype in up to 96,034 individuals with objective accelerometer based measures, related to experimental procedures.

Accelerometer derived activity trait	BETA	SE	P
Proportion of activity over 100mg (moderate to vigorous)	-0.00769687	0.00468483	0.098
Proportion of activity over 40mg (non sedentary)	-0.0101416	0.00468731	0.029
Proportion of activity under 40mg (non sleeping sedentary)	0.0104298	0.00468728	0.025
Midpoint sleep*	0.0114798	0.00494578	0.023

*based on timing of average of least active 5 hours during 24 hour daily cycle.
